# AI in endodontics: enhancing precision, efficiency, and personalized care

**DOI:** 10.1097/MS9.0000000000004385

**Published:** 2026-01-20

**Authors:** Omer Abdullah, Marhaba Saleem, Zunaira Hussain, Hamza Mukhtiar, Khushi Ochani, Sidhant Ochani, Kaleem Ullah, Md. Asaduzzaman Nur

**Affiliations:** a Department of Dentistry, Dow University of Health Sciences, Karachi, Pakistan; b Department of Medicine, Khairpur Medical College, Khairpur, Pakistan; c Department of Hepatobiliary Surgery, Al-Amiri Hospital, Kuwait; d Faculty of HPB Surgery, Enam Medical College, Dhaka, Bangladesh

**Keywords:** Artificial Intelligence, deep learning, diagnostic imaging, endodontics, root canal treatment

## Abstract

Artificial Intelligence (AI) is shifting oral health care from a hardware-centric to a software-centric approach, with the potential to solve problems similarly to humans. It is transforming endodontics by enhancing diagnostic accuracy, improving treatment planning, increasing clinical efficiency, and supporting personalized care. This review explores AI applications in endodontics, including working length determination, periapical and caries detection, complex root anatomy segmentation, vertical root fracture identification, and surgical planning. AI models such as convolutional neural networks and artificial neural networks demonstrate high accuracy in interpreting cone-beam computed tomography and radiographic data. Benefits include improved workflow efficiency, reduced misdiagnoses, and individualized treatment strategies. However, challenges persist, such as limited high-quality datasets, regulatory gaps, and ethical considerations. Future directions emphasize technological advancements, collaborative AI-clinician integration, and AI-driven education and training tools. AI stands as a transformative force in endodontics, requiring responsible implementation and continued research to optimize its impact on patient care and clinical practice.

## Introduction

Artificial Intelligence (AI) is shifting oral health care from a hardware-centric to a software-centric approach, with the potential to solve problems similarly to humans. It not only aids in accurate diagnosis but also assists in microsurgeries, treatment planning, documentation, and teaching undergraduate students. It can already detect anatomy better than humans. The real-world usefulness of AI depends on its ease of use, processing speed, and the amount of data it can learn from to build trust. Endodontics, a field majorly dependent on image interpretation, benefits significantly from the integration of AI in various areas. It is important to emphasize that AI is intended to enhance and expand existing capabilities in endodontic practice, not replace the expertise of clinicians. This study has been reported in line with the TITAN guidelines 2025. ^[1]^

**What we already know:**

- Artificial Intelligence (AI) is becoming more common in dentistry, especially for diagnosis and treatment planning.
- Dentists usually rely on radiographs and their own experience to detect problems in root canals.
- Cone-beam computed tomography scans give more detailed images but take time and cost more.

**What this articles adds:**

- Shows how AI can detect root canal anatomy, fractures, and infections more accurately than traditional methods.
- Describes how AI models like convolutional neural networks help in identifying difficult tooth structures and planning treatments.
- Explains how AI is being used in surgeries with tools like augmented reality and robotics.
- Points out that while AI tools are promising, more research and data are needed before they are widely used in clinics.

## Background of endodontics

Endodontics deals with diseases related to the pulp and periapical tissues, as well as conditions affecting the root canal complex, often resulting from dental carious lesions or trauma. These are commonly managed through nonsurgical root canal treatment (RCT)^[2]^. The significance of endodontics lies in its crucial role in preserving tooth structure, function, and providing pain relief. Diagnosis in endodontics primarily relies on an accurate understanding of the disease, its progression in the tooth and surrounding structures, and radiographic imaging for treatment planning.

## The role of technology in dentistry

AI is the capability of a machine to learn from data and mimic human intelligence through problem-solving and decision-making. AI plays a significant role in effective health care by enhancing diagnostic precision and reducing variability in clinical assessments. In recent years, it has demonstrated strong potential to advance modern dentistry by minimizing diagnostic errors and improving treatment planning. AI has shown greater accuracy, efficiency, and reliability in detecting attachment loss and alveolar bone loss compared to diagnoses made by senior specialists, specialists, and general dentists^[3]^. It can also assist dental practitioners in making accurate diagnoses when a second opinion is needed or time is limited.

## AI in diagnosis

Intraoral periapical radiographs (IOPA), orthopantomograms, and cone-beam computed tomography (CBCT) are the most commonly used radiographic techniques for diagnosing diseases related to the pulp and periapical areas^[2]^. CBCT, which generates three-dimensional images with greater precision, offers improved accuracy in detecting periapical lesions^[2]^. However, periapical and panoramic radiographs, which produce two-dimensional (2D) images of the maxillofacial structures with lower radiation exposure, are often more advantageous. The use of CBCT is typically reserved for specific clinical situations where its benefits outweigh the potential risks associated with its higher radiation dose^[2]^.

## AI applications in endodontics

### Working length determination

Working length must be accurately measured for RCT to be successful. Radiography and electronic apex locators are the most commonly used techniques to determine working length. Artificial neural networks (ANNs) enhance the precision of working length determination by locating the position of the apical foramen on a radiograph. ANNs provide more precise working length measurements than endodontist analysis using IOPA. ANNs have been reported to significantly outperform endodontists in identifying minor anatomic constrictions using IOPA. Therefore, the application of ANNs is considered a reliable method for calculating working length^[4,5]^.

### Periapical lesion detection

CBCT interpretations are more accurate than radiographs; however, they are time-consuming and may be overlooked by clinicians. AI, through deep learning, can assist clinicians in diagnosing periapical pathologies more accurately. AI-based detection of periapical lesions using periapical radiographs and CBCT volumes has been shown to be equivalent to or even superior to diagnoses made by experienced specialists^[6]^.

### Caries detection

Numerous studies have evaluated the efficacy of AI models, including convolutional neural networks (CNNs) and ANN algorithms, for the diagnosis, classification, segmentation, and prediction of dental caries. The most commonly used AI algorithms include multilayer perceptron, support vector machines, and neural networks, particularly CNNs. CNNs have demonstrated high specificity, sensitivity, and area under the curve values in detecting dental caries. Neural networks trained using backpropagation (an algorithm for training ANNs) have achieved accuracy rates of up to 99% on dental image datasets. AI-assisted analysis of panoramic radiographs helps dentists detect caries accurately, with a precision rate of 91.5%. AI can also help stabilize costs and improve affordability by reducing the number of undetected lesions. The ResNet50-DCDNet model outperformed all other models by correctly identifying occlusal and proximal caries, achieving the highest F1 score of 62.7%. CariesNet, a CNN-based model designed for interpreting dental radiographs, reached an impressive accuracy rate of 93.61% in detecting dental caries, surpassing traditional manual inspections^[7]^.

### Automated detection of canal system

AI has proven effective in detecting second mesiobuccal canals in maxillary molars, C-shaped configurations in mandibular molars, and pulp calcifications. Anatomical detection is valuable in minimizing errors, as it helps reduce the risk of unintended harm and injury to surrounding anatomical structures^[8]^.

### Complex root morphology and anatomy detection

The application of AI in endodontics has significantly improved the prediction of complex root canal structures, including C-shaped canals and accessory MB2 canals. CNNs, trained on CBCT data, have advanced the recognition of these complex anatomies. A review by Albitar *et al*^[9]^ suggests that AI architectures such as CNNs can be optimized to automatically detect and segment unobturated canals in endodontically treated teeth. Similarly, Jeon *et al*^[10]^ concluded that deep learning models effectively detect C-shaped canals in mandibular second molars. Despite this progress, challenges persist, such as the limited availability of training data for rare anatomies and the “black box” nature of CNNs, which can reduce clinical confidence^[11]^. Technologies like super-resolution methods and standardized protocols are essential to bridge these gaps. However, comprehensive and heterogeneous datasets are still needed to minimize diagnostic errors.

## Comparative overview of AI models in endodontics

Recent developments in AI have introduced advanced deep learning models into endodontic diagnostics. These models are designed to assist in detecting complex anatomical structures and pathologies with high accuracy. Table 1 provides a summary of validated AI models, their applications, imaging modalities, and diagnostic performance, as reported in selected studies^[9,10,12]^. These studies demonstrate that AI models such as U-Net and Xception CNN exhibit strong diagnostic capabilities in endodontics. CBCT-based models offer high sensitivity and accuracy for anatomical segmentation, while 2D panoramic models like Xception achieve excellent results in predicting root canal morphology. The consistent performance across different imaging modalities suggests AI’s versatility and potential to support clinicians in both routine and complex cases.

**Table 1:** AI models used in endodontics with their respective applications, imaging modalities, and reported performance metrics based on cited studies

Study (Year)	AI model	Application	Imaging modality	Performance metrics
Albitar *et al* (2022)^[9]^	U-Net CNN	Detecting unobturated MB2 canals	CBCT	Sensitivity 0.80, specificity 1.00, and accuracy 0.90
Jeon *et al* (2021)^[10]^	Xception CNN	Predicting C-shaped canals	Panoramic radiographs	Accuracy 95.1%, sensitivity 92.7%, specificity 97.0%, and precision 95.9%
Sherwood *et al* (2021)^[12]^	Residual U-Net, Xception U-Net	Segmentation of C-shaped canals	CBCT	Dice coefficient: Xception 0.768, Residual 0.736, U-Net 0.660

### Vertical root fracture detection

AI systems are enhancing the detection of vertical root fractures (VRFs), which are often missed in traditional radiographs due to overlapping structures or subtle cracks. A study by Abdelazim *et al* evaluated the effectiveness of AI in identifying root fractures on 2D periapical radiographs using a voting system involving five different algorithms to address model discrepancies. The AI-based system showed high precision and sensitivity in detecting root fractures^[13]^. However, 2D radiographs lack the 3D detail offered by CBCT scans, which are more accurate but costly and less accessible^[14]^. AI models also require large and diverse datasets to minimize bias, yet current training data remain limited^[15]^. Future research should prioritize clinical trials using natural fractures and incorporate 3D imaging to improve reliability^[13]^.

### AI in endodontic surgery

AI is aiding endodontists in enhancing surgical accuracy and outcomes. AI models can analyze CBCT scans to detect periapical lesions and root fractures more reliably than humans, thereby reducing observer bias^[16]^. It also assists in identifying root canal anatomy and critical structures such as the inferior alveolar nerve, which is essential during surgical procedures^[16]^. AI supports decision-making by predicting treatment outcomes, guiding dentists in choosing between surgical and nonsurgical retreatments^[17]^. Augmented reality combined with AI is being utilized in guided surgeries like apicoectomies, improving accuracy and minimizing complications^[18]^. Real-time support tools are in development to help clinicians accurately identify the working length and apical foramen^[16]^. AI also aids in planning minimally invasive surgical paths, helping avoid damage to vital structures^[17]^. In robotic-assisted endodontic microsurgery, AI enables precise movements using real-time data, ensuring safer and more consistent procedures^[18]^. However, most AI tools remain in the research phase and are not yet widely adopted in clinical settings. Clinical validation, standardization, and ethical frameworks must be strengthened before AI becomes routine in endodontic surgery. Future research should focus on building diverse datasets and validating AI models across varied populations to reduce bias and enhance accuracy.

## Benefits of AI

### Enhancing diagnostic accuracy

AI has revolutionized endodontic diagnostics by enhancing the precision in identifying complex pathologies. AI-driven tools, such as CNNs, achieve up to 93% accuracy in detecting VRFs via CBCT, surpassing traditional radiography methods^[16]^. Automated systems also demonstrate high performance in tracing root canal morphology, achieving up to 95.1% accuracy, which significantly reduces human error in anatomical assessments^[10]^. Moreover, AI improves prognostic evaluations – such as predicting the success of retreatments – by integrating clinical and radiographic data, enabling more evidence-based decision-making^[19]^. These advancements streamline workflows, minimize misdiagnoses, and improve treatment outcomes in endodontics.

### Efficiency and time saving

AI optimizes endodontic workflows by automating time-consuming diagnostic tasks with high accuracy. AI algorithms, such as ANNs, reduce procedural time by accurately locating the apical foramen (96% accuracy), compared to manual methods (76% accuracy), thereby minimizing the need for repeated measurements^[20]^. Deep learning models can detect periapical lesions on CBCT scans with 93% accuracy in seconds, eliminating lengthy manual evaluations^[21]^. AI also speeds up root fracture detection and treatment planning, reducing chairside time without compromising reliability^[16]^. These advancements enhance clinical efficiency and allow practitioners to focus more on complex decision-making and patient care.

### Providing personalized approaches to individuals

AI significantly enhances personalized endodontic treatment by tailoring plans to the clinical variations of individual patients. AI algorithms incorporate patient-specific data – such as CBCT images, genetic profiles, and medical histories – to predict outcomes like retreatment success^[21]^. For example, deep learning algorithms effectively manage diverse root canal anatomies, including the segmentation of C-shaped canals^[16]^. Additionally, AI improves risk assessment by thoroughly analyzing radiographs and offering evidence-based treatment recommendations based on patient risk profiles^[12]^. These advanced capabilities ensure that treatments are both personalized and clinically accurate.

## Challenges and limitations of AI in endodontics

### Data availability and quality

The success of AI largely depends on the availability and quality of data used for training and validation. Without high-quality data, AI cannot reach its full potential. Despite the availability of data, significant limitations persist. Publicly accessible databases for endodontic radiographs (e.g., CBCT, periapical radiographs) remain limited. Additionally, datasets are often biased toward common pathologies, leading to underrepresentation of rare conditions. Unlike other medical specialties that have adopted open-source AI platforms, dentistry – particularly endodontics – lacks centralized repositories and validated models. Emerging solutions like federated learning and cloud-based AI platforms have been proposed to enhance data access and model training^[22]^.

### Regulatory and ethical concerns

The implementation of AI in endodontics raises substantial regulatory and ethical challenges that must be addressed to ensure patient safety and equitable care. Comprehensive regulatory guidelines are essential to govern the development and use of AI systems, ensuring adherence to quality and safety standards. The absence of universal regulations for AI tools in endodontics results in inconsistencies in performance, data quality, and patient safety. AI tools must be evaluated and approved by authorities such as the U.S. Food and Drug Administration (FDA) or European Medicines Agency (EMA)^[8]^.

Since AI relies on radiographic and patient history data, compliance with data protection laws is critical. Furthermore, clear guidelines regarding liability and accountability are necessary to address potential AI-related errors and ensure fair compensation for patients and responsibility for clinicians^[23]^.

Transparency in AI decision-making is essential to build trust between patients and health care providers. As AI technology evolves, clinicians must be trained not only in its effective use but also in understanding its ethical implications. Responsible data handling and patient engagement can help mitigate algorithmic bias and improve health care quality for a broader population^[24]^.

### Integration with current endodontic practices

Integrating AI into endodontic practice requires a detailed assessment of existing workflows. Clinical data mining methods offer insights into treatment effectiveness and outcomes, allowing practitioners to tailor AI tools to specific clinical needs and patient populations^[25]^. As AI systems advance, it is also essential to develop training programs that include both simulated and real-world applications. These approaches streamline clinical operations, improve patient care quality, and contribute to a more adaptable and equitable health care environment.

## Future directions of AI in endodontics

### Technological advancements

Future AI developments are expected to reshape endodontics by enhancing diagnostic precision, treatment planning, and patient outcomes through custom algorithms and data-driven insights^[12,19–27]^. CNNs already support the diagnosis of periapical lesions, predict treatment outcomes, and generate photorealistic 3D reconstructions of tooth anatomy^[28]^. Additionally, predictive analytics can improve planning, while machine learning continues to enhance diagnostic accuracy. AI’s procedural support enables clinicians to deliver more efficient and effective care^[27]^.

### Collaborative roles of AI and endodontists

AI and endodontists can work collaboratively by combining data-driven insights with clinical expertise to improve patient outcomes. AI can detect complex root canal anatomies, analyze radiographic images, and identify periapical pathologies that may go unnoticed by the human eye. Leveraging large datasets, AI can offer predictive analytics and evidence-based treatment suggestions, empowering endodontists to make more informed decisions tailored to each patient. Additionally, AI tools can assist in documentation, provide real-time guidance, and help clinicians focus on patient care and procedural accuracy^[6]^.

### Impact on education and training

AI is not intended to replace human expertise but to serve as a valuable tool for enhancing precision, productivity, and consistency. In dental education, AI supports undergraduate training through AI-powered simulation platforms and virtual reality tools, enabling students to practice procedures in a safe environment and develop hand skills^[29]^. AI also promotes lifelong learning by offering data-driven self-assessment tools, performance tracking, and personalized learning modules that address individual knowledge gaps. Overall, AI is becoming a transformative force in shaping the future of dental education^[30]^.

## Conclusion

AI is reshaping endodontics by enhancing diagnosis, treatment planning, and patient care. It also supports education through personalized learning and simulation tools. Despite these benefits, challenges persist – including limited data quality, lack of standardized guidelines, and ethical concerns. Addressing these issues requires workflow adaptation and practitioner training. Moving forward, the focus should be on expanding educational tools, fostering collaboration between AI and clinicians, and advancing diagnostic technologies.

## Data Availability

Available upon reasonable request from the corresponding author.
